# Evaluating the early diversification of *Yersinia pestis* and its phylogeographic expansion over 96 years of evolution in Madagascar

**DOI:** 10.1038/s42003-025-09109-1

**Published:** 2025-11-26

**Authors:** Lovasoa Nomena Randriantseheno, Jason W. Sahl, Adrien Rieux, Guillem Mas Fiol, Dawn Birdsell, Olivier Gorgé, Eric Valade, Javier Pizarro-Cerdà, Voahangy Andrianaivoarimanana, David M. Wagner, Minoarisoa Rajerison

**Affiliations:** 1 Plague Unit, Institut Pasteur de Madagascar, Antananarivo, Madagascar; 2 Ecole doctorale Sciences de la Vie et de l’Environnement, University of Antananarivo, Antananarivo, Madagascar; 3 The Pathogen and Microbiome Institute, Northern Arizona University, Flagstaff, AZ USA; 4 CIRAD, UMR PVBMT, F-97410 St Pierre, La Réunion, France; 5 Institut Pasteur, Université Paris Cité, CNRS UMR6047, Yersinia Research Unit, Paris, France; 6 Institut de Recherche Biomédicale des Armées, Brétigny-sur-Orge, France; 7 Institut Pasteur, French National Reference Laboratory ‘Plague & Other Yersiniosis’, WHO Collaborating Centre for Plague, Paris, France; 8 The Biodiscovery Institute, The University of Nottingham, Nottingham, UK

**Keywords:** Phylogenetics, Bacterial genetics, Pathogens

## Abstract

Madagascar is the most plague-affected country globally, yet the phylogenetic diversity of *Yersinia pestis* in this country remains insufficiently characterized. In this study, we analyzed whole-genome sequences of 614 *Y. pestis* strains, with 141 strains newly sequenced, collected over 96 years across Madagascar. All isolates emerged in 1898 and belong to the phylogroup 1.ORI3, supporting a single introduction event to Madagascar, followed by local diversification. We identified 23 distinct subgroups, including eight previously undescribed. Although most novel subgroups were rarely detected or rapidly extinguished, the ɣ subgroup circulated between 2016 and 2019 in southeastern Madagascar—an area historically plague-free for 64 years. Our analysis revealed extensive strain diversity and subgroup persistence, with some subgroups, such as α and β, persisting silently for decades before reemerging during the 2017 pneumonic plague epidemic. These findings uncover deep, previously underestimated phylogenetic diversity and long-term dynamics of *Y. pestis* in Madagascar, providing critical insights for understanding transmission patterns and informing future plague surveillance and control efforts.

## Abstract (web summary)

A comprehensive phylogenetic analysis of *Yersinia pestis*, the causative agent of plague, provides insight into the diversity and evolution of this pathogen in Madagascar. This study provides valuable information for understanding transmission and outbreak dynamics in the region.

## Introduction

*Yersinia pestis* is one of the most feared pathogens in history. It is known to have evolved from the enteropathogen *Y. pseudotuberculosis*, perhaps as recently as 7400 years ago^1^. Despite this evolutionary link, *Y. pestis* causes plague, a disease with vastly different clinical manifestations. Three historical plague pandemics were recorded in human history, with the third pandemic responsible for the worldwide spread of the disease following the advent of steamships. It is thought that a steamship from India introduced plague to Madagascar at the port city of Toamasina in 1898^2^. For the next decade, other port cities reported cases and, in 1921, plague reached the capital city of Antananarivo, located in the Central Highlands, where an epidemic occurred^2^. From the capital, the disease spread to other regions, leading to the establishment of plague as an endemic disease in the Northern and Central Highlands of Madagascar (areas located at an elevation ≥800 m) and in the port city of Mahajanga^3^. Nowadays, hundreds of human cases are declared in known plague foci in the Northern and Central Highlands each year. As recently as 2017, a major epidemic of pneumonic plague occurred in urban cities, including Antananarivo. These recent epidemics reminded us of the continuing threat posed by the disease^4,5^.

A single population of *Y. pestis* (1.ORI) caused the third plague pandemic^6^, of which a single subpopulation (1.ORI3) is present in Madagascar^6,7^. Previous efforts to genotype the 1.ORI3 subpopulation of Madagascar revealed a surprisingly high level of diversity with 18 major subgroups (13 subgroups identified via SNPs analysis and 5 subgroups identified via multi-locus variable number tandem repeats analysis-based). These major subgroups exhibited geospatial structure (i.e., subgroups endemic to specific geographic locales), and this structure may be linked to topography^7^. Uncovering this spatial structure has enabled further investigation into the dynamics of the disease, including *Y. pestis* dispersal events, and the evaluation of the persistence of various subgroups. The knowledge gained from these studies could ultimately help to control and prevent plague activity in Madagascar and elsewhere. However, due to the limited number of sequenced Malagasy *Y. pestis* strains, the real phylogenetic diversity is likely still underestimated. In this study, we analyzed whole-genome sequences of 614 *Y. pestis* strains from Madagascar, revealing eight lineages that, to our knowledge, had not been described before. These findings support previous hypotheses while expanding the known phylogenetic diversity of *Y. pestis* in the region. Additionally, we provide a more recent estimate of the emergence of different subgroups and offer insights into the geographical distribution and persistence patterns of these identified genotypes.

## Results and Discussion

In this study, we generated and analyzed an extensive whole-genome dataset for *Y. pestis* in Madagascar spanning 96 years (1926–2022). Since its introduction to Madagascar in 1898, *Y. pestis* has become endemic, causing seasonal outbreaks each year^3,8–10^ and we aimed to trace its evolutionary history from introduction to the current distribution across various plague foci. For that, we generated whole-genome sequences for 141 *Y. pestis* strains and then, a maximum likelihood phylogeny for a total of 614 strains from Madagascar using 919 SNPs (Supplementary Data 1). Some of the earliest-isolated *Y. pestis* strains from Madagascar were included in this study allowing us to have insights into the early diversification of *Y. pestis* soon after its introduction to Madagascar. To our knowledge, this study presents the largest phylogeny of Malagasy *Y. pestis* that is constituted exclusively of whole genome sequenced strains. The TempEst results (Supplementary Fig. 1) showed a low R² (R² = 0.1232) reinforcing the deviation from strict molecular clock, which is already well known in *Y. pestis*^11^. However, the period during which the strains were isolated allowed us to estimate the time to the most recent common ancestors (TMRCAs) of the newly identified subgroups, and also to update the estimate made for each subgroup in a previous study, which was based on only 33 strains^7^. Of note, our sampling was opportunistic and uneven across space and time (Supplementary Fig. 2). As such, we are being conservative about the conclusions that we can draw from this dataset, but several major patterns can be observed.

**Fig. 1:**
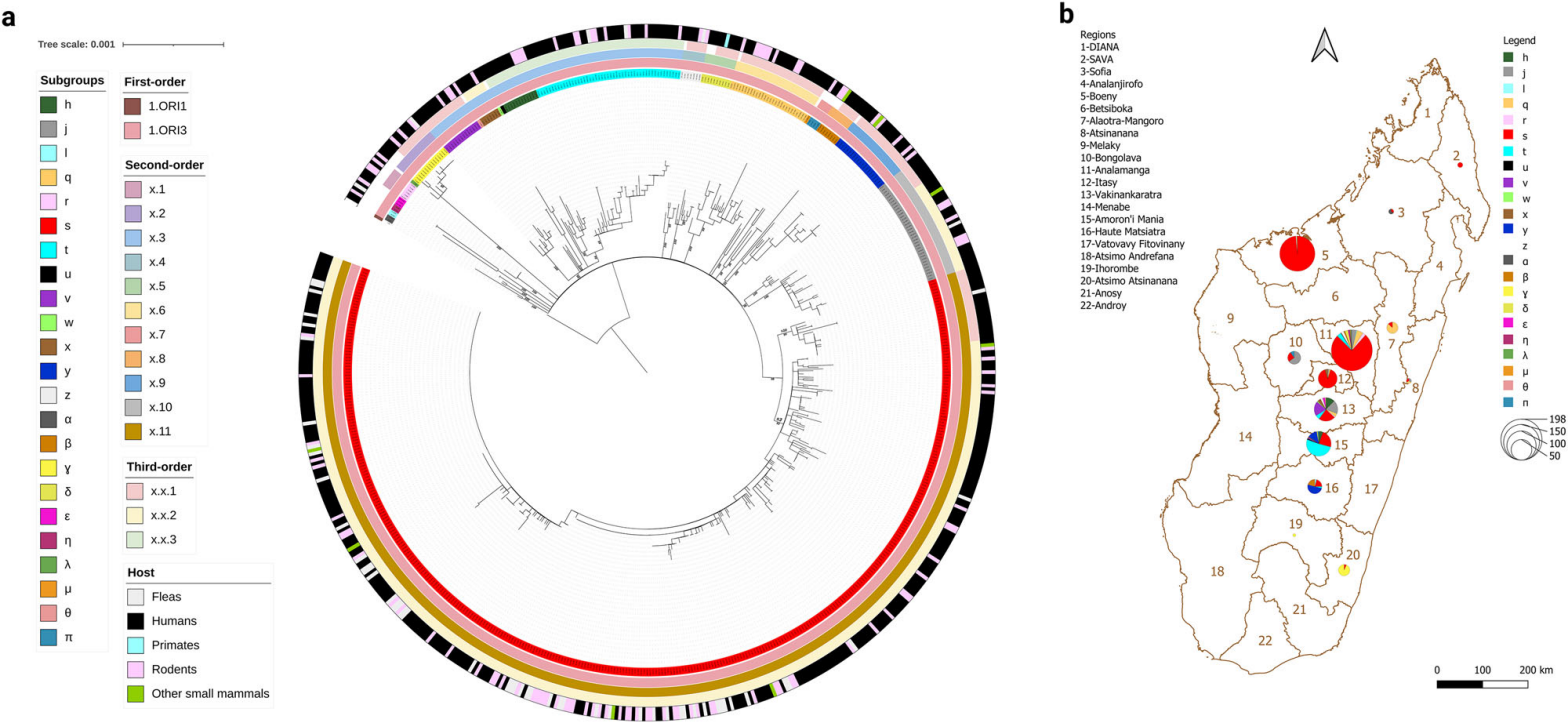
Phylogeography of *Y. pestis* in Madagascar. **a** Maximum-likelihood phylogeny of Malagasy *Y. pestis* strains rooted on strain CO92 (isolated in the USA). The tip labels (first circle from the center) are colored according to the color of the subgroup of the strains identified in this study via whole-genome sequencing. The second, third and fourth circles from the center represent the different levels (first, second and third-order) of the hierarchical nomenclature of *Y. pestis* as established by Wu et al. ^39^. Colors of the fifth circles represent the host from which the strain was isolated. Numbers on branches represent bootstrap values. **b** Map of the distribution of the different phylogenetic subgroups across the regions of Madagascar. The pie charts represent the number of *Y. pestis* strains isolated from a given region and the colors represent the different subgroups (source data is presented in Supplementary Data 5). The subgroup colors are the same as in Vogler et al. ^7^.

**Fig. 2:**
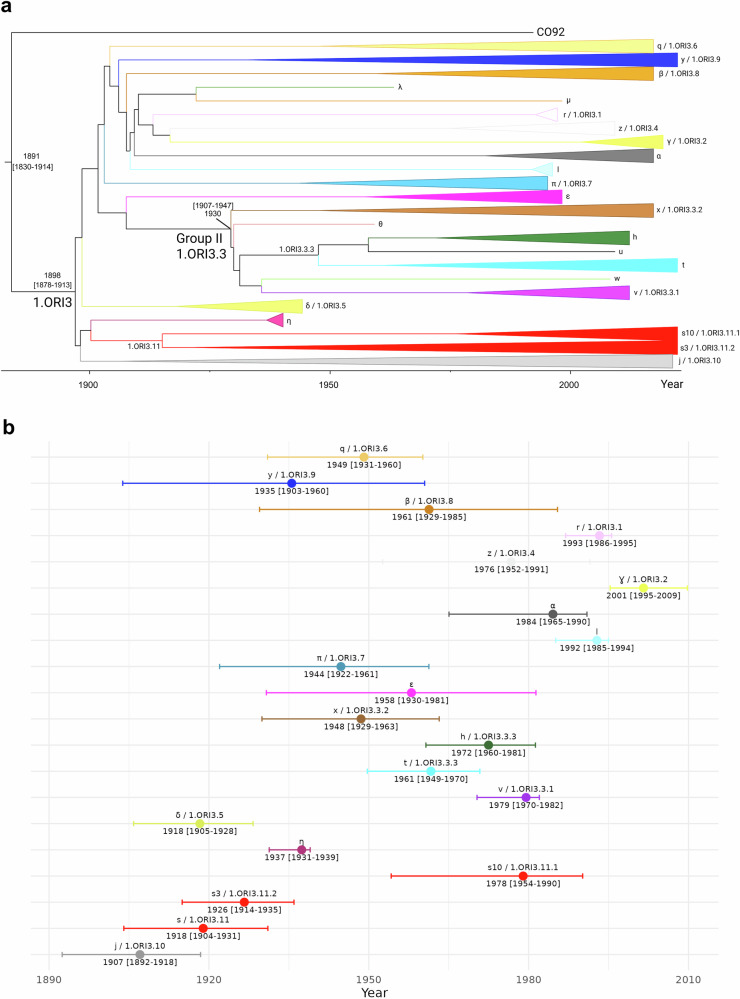
Molecular dating of the 614 *Y. pestis* strains from Madagascar. **a** Maximum clade credibility tree rooted on *Y. pestis* CO92. Each subgroup is collapsed and colored with the same colors as in Fig. 1. **b** Representation of the median of the most recent common ancestors of each subgroup (excluding the five subgroups containing only one strain) with their 95% highest posterior density as error bars (source data are presented in Supplementary Data 6-7) colored with the color of the corresponding subgroup.

Our results are consistent with a single introduction of *Y. pestis* into Madagascar during the third pandemic followed by a rapid radiation. The observation of the star-like shape of the phylogeny, similar to previous reports^6^, and the fact that all the strains belong to the monophyletic branch 1.ORI3 (Fig. 1a), which is only found in Madagascar and Turkey, further supports this hypothesis. This pattern is similar to that observed with *Y. pestis* in Brazil^12^ and, in fact, a star-like phylogeny has been observed repeatedly in the evolution of *Y. pestis* and at multiple spatial scales^13^. In our analyses, the deep branches of the phylogenetic tree are well supported with bootstrap values ≥ 95% (Fig. 1a). The median substitution rate of 4.024×10^-8^ substitutions per site per year (95% HPD (highest posterior density): [3.395×10^-8^ - 4.720×10^-8^]) is similar to the results reported previously^14^. The median TMRCA of all the *Y. pestis* strains from Madagascar was estimated to be in 1898 (95% HPD: [1878, 1913], mean: 1896) (Fig. 2a), coinciding with the year plague was introduced to Madagascar. These findings suggest the emergence of 1.ORI3 immediately following the introduction of plague in Madagascar, whereas previous studies estimated it later (in 1954^7^ or 1905^14^). However, we cannot rule out the possibility of more than one introduction event. Evidence for such possible events is lacking in the data presented here, but it is possible that lineages resulting from separate introductions underwent early extinction or were not isolated, knowing that only a few early strains have been studied.

Soon after being introduced to Madagascar in 1898, *Y. pestis* rapidly differentiated into multiple distinct phylogenetic groups as evidenced by the TMRCAs of the different subgroups estimated here in our study (Fig. 2) and in previous studies^7,14^. This was likely facilitated by the fact that *Rattus rattus*, estimated to have been introduced to Madagascar in the 10^th^ century^15,16^ or before^17^, had already disseminated extensively across the island by that time, establishing a conducive environment long before the arrival of *Y. pestis*. Once introduced, the widespread presence of *R. rattus* likely facilitated the persistence of *Y. pestis* within local rat populations for extended periods. This prolonged interaction probably led to the differentiation of local populations, the establishment of multiple distinct environmental foci, and a strong correlation between geographic locations and the phylogenetic groups of *Y. pestis*^7^. However, this geographical structure of phylogroups might also be due to genetic drift resulting from the founder effect when a particular group is introduced into a new area. In addition, although not examined here, it has previously been indicated that plague dispersal between foci followed by ecological establishment is rather rare^7^, further explaining the distinct phylogenetic character of the different geographical foci. These stable environmental foci, located throughout the central highlands, help explain why the majority of human cases are predominantly confined to these regions^18^.

**Fig. 3:**
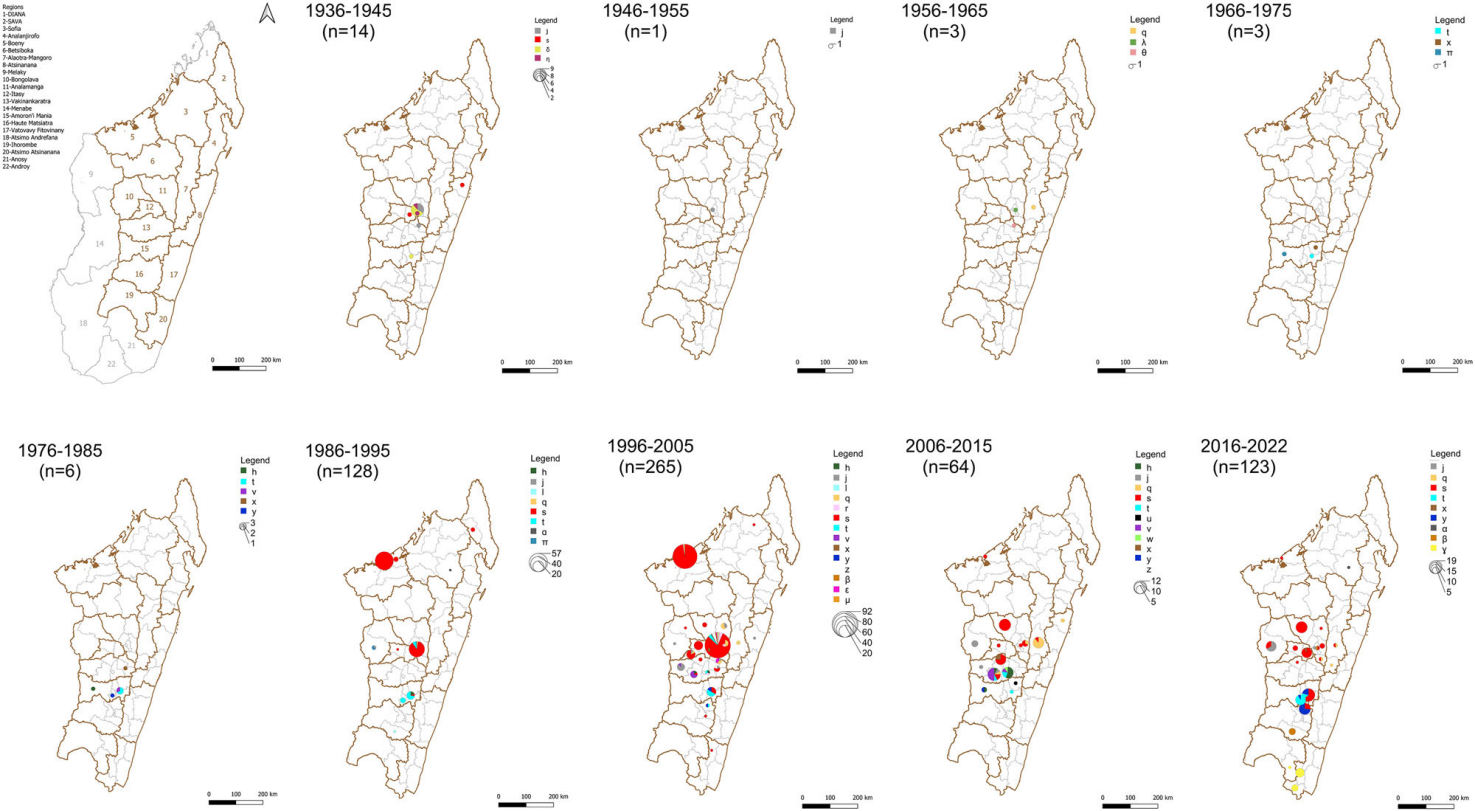
Phylogeographic distribution of the 614 strains of *Y. pestis* in the districts of Madagascar. In the first map, all 22 regions of Madagascar are presented (gray: regions not represented in the remaining maps, brown: regions represented in the remaining maps). In the remaining maps, districts are represented as thin black lines inside each region. Districts are an administrative subdivision within the regions in Madagascar; the number of districts grouped in a region is variable. The pie charts represent the number of *Y. pestis* strains isolated from a given district and each color represents a subgroup as detailed in the legends (source data is presented in Supplementary Data 3).

Our analyses revealed the long-term persistence (≥25 years) of multiple *Y. pestis* phylogenetic groups in the Central Highlands regions of Madagascar. For instance, the j subgroup has persisted in the Central Highlands for the longest time, almost 100 years (Fig. 3), but other phylogenetic subgroups have persisted for at least 25 years, namely s (isolated from 1941–2022), q (1962–2017), x (1969–2017), t (1974–2022), v (1982–2012), and h (1984–2012). Persistence of some of these phylogenetic groups for ~20 years has already been reported previously^7^ but here we demonstrate temporal persistence well beyond that. Plague persistence is largely limited to the highland regions (≥800 m elevations) where ecological conditions are suitable for a flea vector, *Synopsyllus fonquerniei*^19^. Despite widespread distribution of *R. rattus* and other flea species throughout Madagascar, the strong co-localization of plague persistence and *S. fonquerniei* flea species in the central highlands illustrates the critical role this flea species serves in sustained plague persistence.

Our analysis of a large set of *Y. pestis* isolates from Madagascar, including older historical isolates, revealed the presence of eight phylogenetic groups not previously described, and some of these are now likely extinct. The identification of ɣ, δ, ε, η, λ, µ, θ and π subgroups extends the known diversity of *Y. pestis* in Madagascar and adds to the resolution of its local phylogeny. Five of these subgroups (ɣ, η, ε, δ, π) are strongly supported by a bootstrap value of 100%. Also, three (π, ε and µ subgroups) are rarely encountered as the last strain belonging to each of them was isolated ~27 years ago. Four are likely extinct now as they have not been observed for >60 years: η (isolated just twice, in 1939 and 1940), δ (isolated only from 1940-1944), θ (isolated just once, in 1959), and λ (isolated just once, in 1963) (Fig. 3, Supplementary Data 2 and 3). Given the limited number of isolates collected prior to 1990 that were analyzed in this study (Fig. 3, Supplementary Fig. 2), it is likely that even more lineages, now extinct, emerged during the early radiation of *Y. pestis* in Madagascar. It is important to note that the perceived extinction of these subgroups might only be the result of recent representatives not being sampled in our study. Considering the example of the recently discovered α and β subgroups^4^, particularly, we found that these subgroups did not emerge recently but had persisted locally for a long time before being isolated during the 2017 pneumonic plague epidemic. Indeed, the median TMRCAs of α and β subgroups were estimated in 1984 (95% HPD: [1965–1990], mean: 1981) and 1961 (95% HPD: [1929–1985], mean: 1959) (Fig. 2b), respectively. For the β subgroup, evidence of its persistence for at least 20 years in the reservoir populations was established by two strains isolated in 1997 from small mammals (one *Rattus norvegicus* and one Asian shrew, trapped from Antananarivo (Analamanga region) and Mahajanga (Boeny region), respectively). During the 2017 plague season, this β subgroup emerged in circulation in the Ambalavao district (Haute Matsiatra region)^4^. Regarding the α subgroup, our data suggest that it has persisted in the district of Mandritsara (Sofia region) for more than 26 years.

*Y. pestis* phylogenetic diversity is higher in the Central Highlands compared to less active plague foci. Topographical relief was found to be associated with a greater level of *Y. pestis* diversity^7^, possibly maintained by different, separated rat populations^20^. Apart from the determined endemic foci, cases have also emerged in other areas in Madagascar, like Ambilobe^21^ and Mahajanga^14,22^, that are now considered as natural plague foci due to the proven persistence of plague there. We show, in our dataset, that additional strains have been isolated from other less active plague foci such as Iakora (Ihorombe region, one strain belonging to the ɣ subgroup), Midongy Atsimo, Befotaka (Atsimo Atsinanana region, 14 strains belonging to the ɣ subgroup), and Vondrozo (Atsimo Atsinanana region, one strain belonging to the s subgroup) districts. These unusual foci are characterized by the presence of only one subgroup (as opposed to 14 in Antananarivo Renivohitra and 6 in Ambositra districts (Supplementary Data 2)). This low level of diversity could be the result of the founder effect when only one subgroup was introduced in these districts and therefore, had the opportunity to evolve, circulate and persist. The single introduction of *Y. pestis* into these districts is possibly due to their extreme isolation, resulting from very low levels of anthropogenic activities and trading of goods to and from these districts. Also, the absence of circulation of an established lineage was thought to be a factor influencing the successful establishment of a subgroup in an area^7^. But, in the case of these districts, we only have evidence of short-term persistence of the described ɣ subgroup since all the strains of Midongy Atsimo, Iakora, and Befotaka districts were isolated in a period of just four years (between 2016 and 2019).

*Y. pestis* may have been silently persisting in the southeast of Madagascar for many years. The long branch of the ɣ subgroup may indicate a long period of adaptation to local conditions such as climate, or potentially new reservoirs or vectors. Indeed, human plague cases were reported from 1949 to 1952 in Midongy Atsimo, which was thought to result from a spread from the Central Highlands^2^. But despite being located at an elevation of 860 m^23^, this area did not regularly report cases in subsequent years. The ɣ subgroup might have evolved in the rodent reservoirs from the strains that started these first outbreaks without spillover to humans, considering that human cases were only reported again in Befotaka and Iakora between 2016 and 2024, and between 2018 and 2024 in Midongy Atsimo. Alternatively, the absence of human cases for 64 years could simply be due to under-reporting resulting from the extreme isolation of these areas, as discussed above, and the lack of access to health care centers, particularly if the human cases occur in locations remote from them. Although the median TMRCA of the ɣ subgroup estimated here is 2001 (95% HPD: [1995–2009], mean: 2001) (Fig. 2b), the split of the z and ɣ subgroups has been dated in 1940 (95% HPD: [1902, 1969], mean: 1938) (Fig. 2a). As explained in a previous study, the actual emergence of a new genotype could have occurred at any time since the separation from its closest relative and along the branch of the emerging new genotype^24^, i.e., at any time between 1940 and the TMRCA of ɣ subgroup dated in 2001, which would support our hypotheses. This estimate would eventually become more accurate if older strains belonging to the ɣ subgroup could be included in future studies, as it is the case for other subgroups here in our study.

Subgroup s is one of the most successful phylogenetic groups, having persisted for at least 81 years (the oldest and most recent strains of this subgroup were isolated in 1941 and 2022, respectively (Supplementary Data 2 and 3)) and were found throughout most plague endemic regions in Madagascar. Its emergence is dated to around 1918 (95% HPD: [1904-1931]) (Fig. 2b). We observed the separation of s subgroup into two major clades thanks to the very extensive sampling of this subgroup (Fig. 1a). The first subclade appears to be restricted to three regions of the Central Highlands foci (Vakinankaratra, Amoron’i Mania, and Haute Matsiatra regions) and is characterized by the SNPs at positions previously identified to separate branch s-s10^7^. This subclade emerged in 1978 (95% HPD: [1954–1990], mean: 1976) (Fig. 2b) and seems to have persisted there for at least 31 years (1991–2022). The second subclade, which emerged in 1926 (95% HPD: [1914–1935], mean: 1925) (Fig. 2b), is characterized by the SNPs previously identified to separate branch s-s3^7^ and is more widespread since all but two (Amoron’i Mania and Ihorombe regions) of the regions sampled here have at least one strain belonging to this clade. The second subclade might also have a higher epidemic potential because strains isolated during the epidemics of the 1990s and 2017 assigned to it and transfers of it between the Central Highlands and Mahajanga were extensively studied^14,22^. Although its tremendous success might be explained at least partially by its age, other reasons, such as potential differences in gene content, could be explored in future studies. In fact, the second subclade of s subgroup is among the earliest-emerged lineages, so it is possible that it spread more during the early radiation occupying empty niches in many locations before other groups. Another hypothesis contributing to the explanation of its frequent isolation could be the fact that it is circulating among rodents in highly populated urban cities, such as Mahajanga and Antananarivo, facilitating its spread after spillover. However, we cannot exclude a bias due to the nature of our samples that were, for the major part, constituted to study the epidemics of the 1990s^14,22^.

There are several limitations to our study. Firstly, the geographical origin of the seven old strains was not recorded, which partially limits the extent of our interpretation. Secondly, overall examination of the phylogeography of *Y. pestis* strains in Madagascar (Fig. 3) has given us the impression that plague foci continue to expand spatially over time. The same hypothesis was emitted when plague reemerged in 1998 after 33 years of silence in the Ikongo district located at an elevation <800 m^25^ but unfortunately, this could not be explored further as no *Y. pestis* strains from this district were included in this study, due solely to the random selection of the strains. However, it is also possible that the plague foci are fairly stable, but our perception is biased by the lack of information resulting from the fact that plague cases are rarely reported from the extremely isolated areas, and that data on more recently isolated strains are much more numerous than that on older strains. Thirdly, the potential differences in gene content among the strains has not been explored. And lastly, this study probably still underestimates the real diversity of *Y. pestis* in Madagascar because we mostly took advantage of sequences obtained from different studies and the number of *Y. pestis* strains sequenced is still low compared to the total number of strains in the IPM’s internal biobank collection. Particularly, only seven strains isolated between 1946 and 1975 were included (Supplementary Data 2 and 3, Fig. 3).

In conclusion, sequencing additional *Y. pestis* strains from Madagascar revealed increased phylogenetic diversity. The persistence of different genotypes in the Central Highlands over a long period, up to almost a century, has been demonstrated, and the possible extension of endemic plague foci through time has been suggested by our results. As plague still represents a major public health problem in Madagascar, efforts to establish infrastructure and technical capabilities needed for pathogen whole genome sequencing must be made to support the local plague surveillance system as well as that of other diseases, and also to rapidly characterize the strains responsible during outbreaks or in the event of potential bioterrorist attacks with antibiotic-resistant strains.

## Methods

### Genomic data and metadata of *Y. pestis* strains

The strains analyzed in this study were isolated between 1926 and 2022 from various plague foci across Madagascar, including the Central and Northern Highlands and Mahajanga, as well as from locations outside the traditionally recognized foci. Most of the genomes included in this study were originally generated for multiple separate studies conducted at the Plague Unit that involved different collaborating institutes (Supplementary Data 2). In total, we used 614 *Y. pestis* genomes with available raw reads, 141 strains were newly sequenced during our study and 78.83% (N = 484) had already been included in multiple published studies^4,7,14,22,26,27^ (although 11 of these had not been sequenced in those previous studies). Among these 614 raw data, 398 were kindly provided by the *Yersinia* research Unit of Institut Pasteur (Paris, France), 75 were downloaded from the Sequence Read Archive (SRA) public database, 11 were sequenced by the Institut de Recherche Biomédicale des Armées (IRBA) team (France), 104 by The Pathogen and Microbiome Institute (PMI) team of Northern Arizona University (NAU, Arizona, USA) and 26 were sequenced in collaboration with the *Yersinia* research Unit and the Cellule d’Intervention Biologique d’Urgence (CIBU) of Institut Pasteur (Paris, France). The metadata associated with these strains, including the year of isolation, district of origin, and host type, were collected (Supplementary Data 2, Supplementary Fig. 3-6). However, the geographical origin of seven strains remains unknown.

### Sample processing

New *Y. pestis* strains sequenced in this study were grown on CIN (Cefsulodin, Irgasan, Novobiocin) plates and in BHI (Brain Heart Infusion) broth for 48 h at 26°C. The pellet in BHI broth was collected and subjected to DNA extraction using QIAamp DNA Mini Kit (Qiagen) following the manufacturer’s protocol, and the colonies grown on CIN plates were used to visually assess culture purity. Different library preparation and sequencing strategies were used at each of the collaborating institute.

### Whole-genome sequencing

For strains sequenced at CIBU, the initial concentration of DNA was checked with the Qubit dsDNA BR Assay Kit. 300 ng of DNA were then used to prepare libraries using Illumina DNA Prep kit (San Diego, California, USA) and Nextera DNA CD Indexes (96 indexes, 96 samples) following manufacturer’s protocol. The concentration of final libraries was checked with Qubit dsDNA HS, then pooled together at equimolar concentrations and quality was assessed using a Fragment Analyzer (Agilent, Santa Clara, CA). Finally, sequencing was done with Illumina MiSeq using the 600 cycles v3 kit (part# MS-102-3003).

For IRBA, libraries were prepared with NEBNext Ultra II DNA Library Prep Kit for Illumina (NEB) and NEBNext Multiplex Oligos for Illumina (96 Unique Dual Index Primer Pairs) following manufacturer’s protocol. Quality assessment of the resulting libraries was done with 2100 Bioanalyzer (Agilent) before sequencing with Illumina NextSeq 500.

For NAU, the genomic DNA (gDNA) extracts were assessed for quality and quantity on a 0.7% agarose gel using λ DNA-HindIII Digest (New England Biolabs, Ipswich, MA, USA). We then sheared the gDNA to ~250 bp using a QSonica Q800 Sonicator (QSonica, Newtown, CT) at 60% amplitude, with 15 s on/off settings. The size of fragments was evaluated using a Fragment Analyzer (Agilent, Santa Clara, CA). WGS library construction was performed using the KAPA Hyper Prep Kits for Illumina NGS platforms per the manufacturer’s protocol (KAPA Biosystems, Woburn, MA, USA, part# KK8504). Sheared samples were end-repaired, A-tailed, indexed with adapter ligation, and amplified. The adapters and 8 bp index oligos purchased from IDT (Integrated DNA Technologies, San Diego, CA, USA) were used in place of those supplied in the KAPA preparation kit. All DNA purification steps were carried out using Agencourt AMPure XP beads (0.8X bead ratio; Beckman Colter, Brea, CA). The final libraries were quantified on an Applied Biosystems QuantStudio 7 Flex Real-Time PCR System using the KAPA SYBR FAST ROX Low qPCR Master Mix for Illumina platforms (part# KK4873). The libraries were then pooled together at equimolar concentrations and quality was assessed using a Fragment Analyzer (Agilent, Santa Clara, CA). Final library quantity was assessed by Qubit Br dsDNA (ThermoFisher Scientific, Waltham, MA). The samples were sequenced on an Illumina MiSeq using the 600-cycle v3 kit (part# MS-102-3003) or Illumina NextSeq 500/550 Mid Output Kit v2.5 (300 cycles) (part# 20024905) used with the standard Illumina procedure.

### Quality check and preprocessing

The quality of the raw reads was checked using FastQC v0.11.9 and results were compiled with MultiQC v1.15^28^ to facilitate visualization. For the reads that needed trimming (Supplementary Data 4), Trimmomatic v0.39^29^ was used with default parameters. Contamination by PhiX reads was also noticed in some of the raw data, so a step to filter PhiX reads was included in the workflow using BBduk, a tool which is part of BBMap v39.01^30^. After trimming and filtering, FastQC and MultiQC were re-run to assess improvements in read quality.

### Downstream processing and analysis

Reads were mapped against the reference genome *Y. pestis* CO92 (GenBank accession no. NC_003143.1) using minimap2 v2.28^31^. Variants were called with GATK v4.1.9^32,33^ using HaplotypeCaller and only included reads with a minimum mapping quality of 30. The resulting VCF files were inputted in NASP pipeline v1.2.1^34^. To identify duplicated regions, the pipeline included the self-alignment of the reference genome using NUCmer v3.1; any SNP that fell within a duplicated region was removed from downstream analyses. Thresholds of 10X and 90% were applied for minimum depth and minimum allele proportion, respectively. SNPs falling in tandem repeat regions or adjacent to another SNP less than 10 bp away were subsequently filtered out from the matrix.

### Phylogeographic analyses

The evolutionary model that best fits the data (TVMe+ASC) was identified with ModelFinder^35^ implemented in IQtree v2.1.2^36^ and used to infer the maximum-likelihood phylogeny with the ultrafast bootstrap (UFBoot^37^, 1,000 replicates) and the SH-like approximate likelihood ratio test (SH-alrT, 1,000 replicates). Visualization and annotation of the resulting phylogenetic tree were undertaken with iTOL v7^38^. The local phylogenetic nomenclature of *Y. pestis* is the main nomenclature used in this study, but the updated hierarchical nomenclature system, developed recently^39^, was also included for a more uniform naming system across studies. For the local nomenclature^6,7^, subgroups were defined based on the branching from the main backbone of the tree, in agreement with the subgroups defined in previous studies^7,22,27^, except for the subgroups in Group II^6,7^. Finally, the geographical distribution of each subgroup was mapped using QGIS v3.22.11.

### Bayesian molecular dating

As a requirement to build tip-calibrated phylogenies, the existence of a temporal signal was investigated using Tempest v1.5.3^40^. Molecular dating was performed using BEAST v1.8.4^41^ by running five Markov Chain Monte Carlo independent replicates of 300 million steps. The xml input file was manually edited to account for the number of constant sites within the alignment. Leaf heights were constrained to be equal to sample ages. Flat priors (i.e., uniform distributions) for the substitution rate (10^-12^-10^-2^ substitutions per site per year) and for the age of all internal nodes in the tree were applied. We also considered a GTR substitution model^42^ with a Γ distribution^43^ and invariant sites (GTR + G + I), an uncorrelated relaxed log-normal clock to account for variations between lineages^44^, and a tree prior for demography of coalescent extended Bayesian skyline^45^. The Bayesian topology was conjointly estimated with all other parameters during the Markov Chain Monte Carlo and no prior information from the tree was incorporated in BEAST. The output files were inspected with Tracer^46^, combined after discarding 10% burn-in. We ensured that the effective samples sizes for key parameters all exceeded 200 before visualizing the maximum clade credibility tree with FigTree v1.4.4.

### Ethics statement

The DNA used in this study were obtained from *Y. pestis* cultures originally isolated from human clinical samples collected by the Central Laboratory for Plague (CLP) and Institut Pasteur de Madagascar (IPM) as part of the Plague National Control Program (PNCP) under the Malagasy Ministry of Public Health. Mandated under PNCP, all suspected human plague cases are reported and biological samples from these cases are collected. These biological samples and any derived isolates or DNA obtained through this compulsory reporting system, were exempt from human subjects research classification and therefore, do not require ethical committee approval with no patient-identifying information associated with them.

### Reporting summary

Further information on research design is available in the Nature Portfolio Reporting Summary linked to this article.

## Supplementary information

Supplementary Information

Description of Additional Supplementary Materials

Supplementary Data [1-6]

Supplementary Data 7

Reporting Summary

## Data Availability

Raw sequence data for all the strains newly sequenced in this study have been deposited in NCBI under Bioproject PRJNA1247195. All accession numbers, for the sequences obtained from previous studies^4,7,14,22,26^ as well as those newly generated, are mentioned in the Supplementary Data 2.
