# Supplementary material for: Evaluating the early diversification of Yersinia pestis and its phylogeographic expansion over 96 years of evolution in Madagascar
Source: Commun Biol. 2025 Nov 26;8:1705. doi: 10.1038/s42003-025-09109-1 (PMC12658076; doi:10.1038/s42003-025-09109-1)
Supplement: Supplementary file 1 — Supplementary Information [file 42003_2025_9109_MOESM1_ESM.pdf]

Evaluating the early diversification of *Yersinia pestis* and its phylogeographic expansion over 96 years of evolution in Madagascar

Lovasoa Nomena Randriantseheno[1,2]*, Jason W. Sahl[3], Adrien Rieux[4], Guillem Mas Fiol[5], Dawn Birdsell[3], Olivier Gorgé[6], Eric Valade[6], Javier Pizarro-Cerdà[5,7], Voahangy Andrianaivoarimanana[1,8#], David M. Wagner[3#], Minoarisoa Rajerison[1#]

[1] Plague Unit, Institut Pasteur de Madagascar, Antananarivo, Madagascar

[2] Ecole doctorale Sciences de la Vie et de l'Environnement, University of Antananarivo, Antananarivo, Madagascar

[3] The Pathogen and Microbiome Institute, Northern Arizona University, Flagstaff, Arizona, USA

[4] CIRAD-BIOS, La Réunion, France

[5] Institut Pasteur, Université Paris Cité, CNRS UMR6047, Yersinia Research Unit, Paris, France

[6] Institut de Recherche Biomédicale des Armées, Brétigny-sur-Orge, France

[7] Institut Pasteur, French National Reference Laboratory 'Plague & Other Yersiniosis', WHO Collaborating Centre for Plague FRA-140, Paris, France

[8] The Biodiscovery Institute, The University of Nottingham, Nottingham, UK

#These authors contributed equally.

*Corresponding author, email: lovasoanomena@pasteur.mg

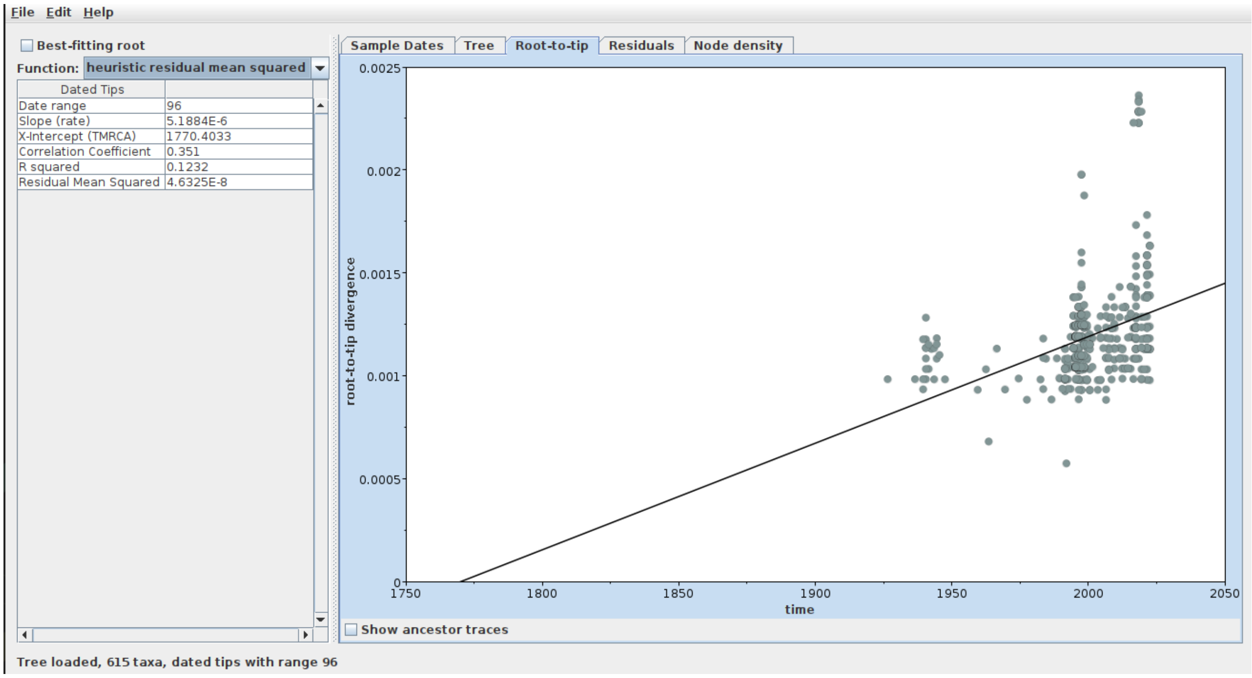

Supplementary Figure 1. **Results of the TempEst assessment of temporal signal in our 614 strains of *Y. pestis* from Madagascar.** The line represents the best-fit linear regression of root-to-tip genetic distance against sampling time.

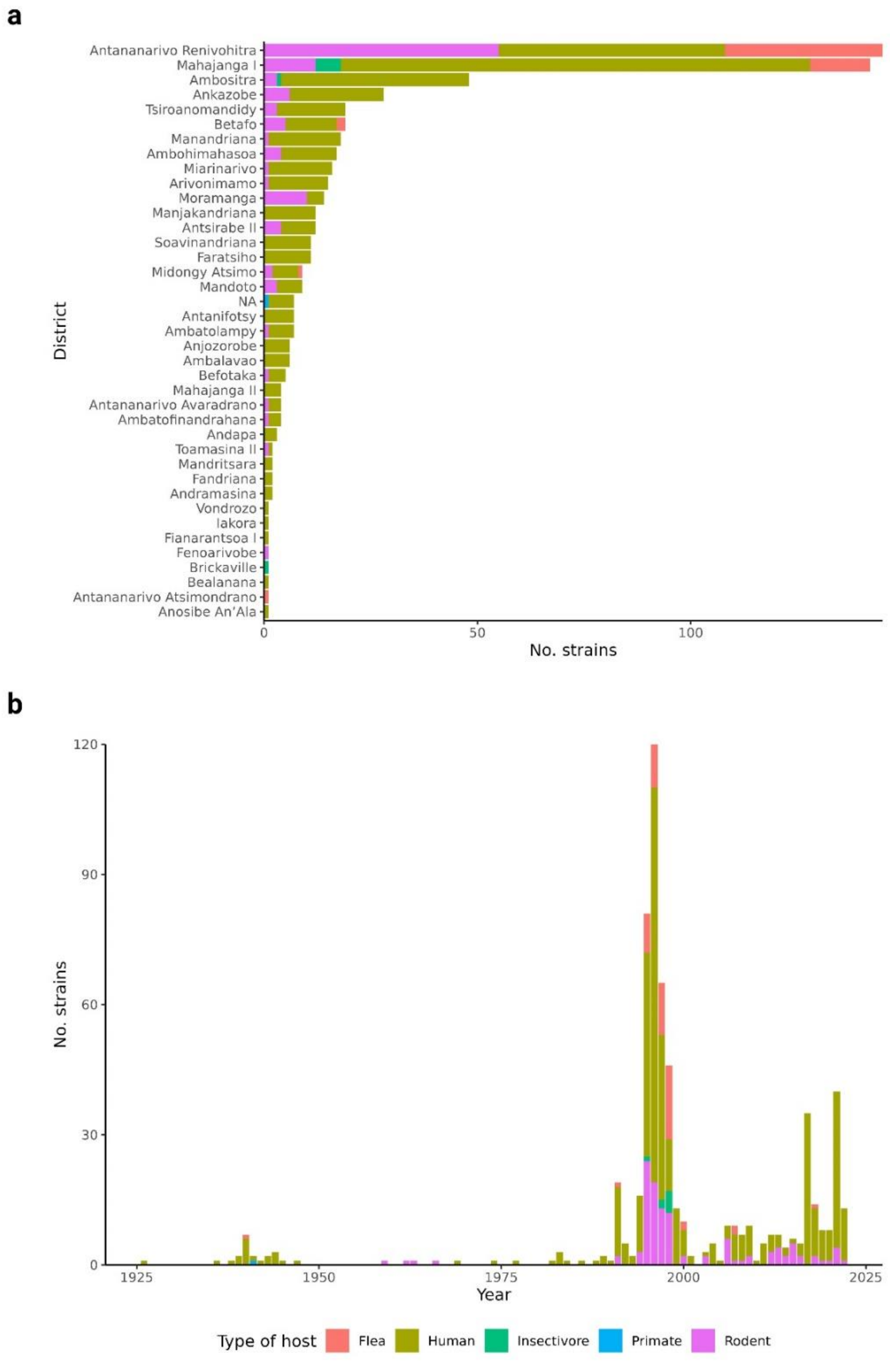

Supplementary Figure 2. **Distribution of the 614 Malagasy *Y. pestis* strains. a** Distribution according to the district of origin (districts are an administrative subdivision within the regions in Madagascar; the number of districts grouped in a region is variable). **b** Distribution according to the year of isolation.

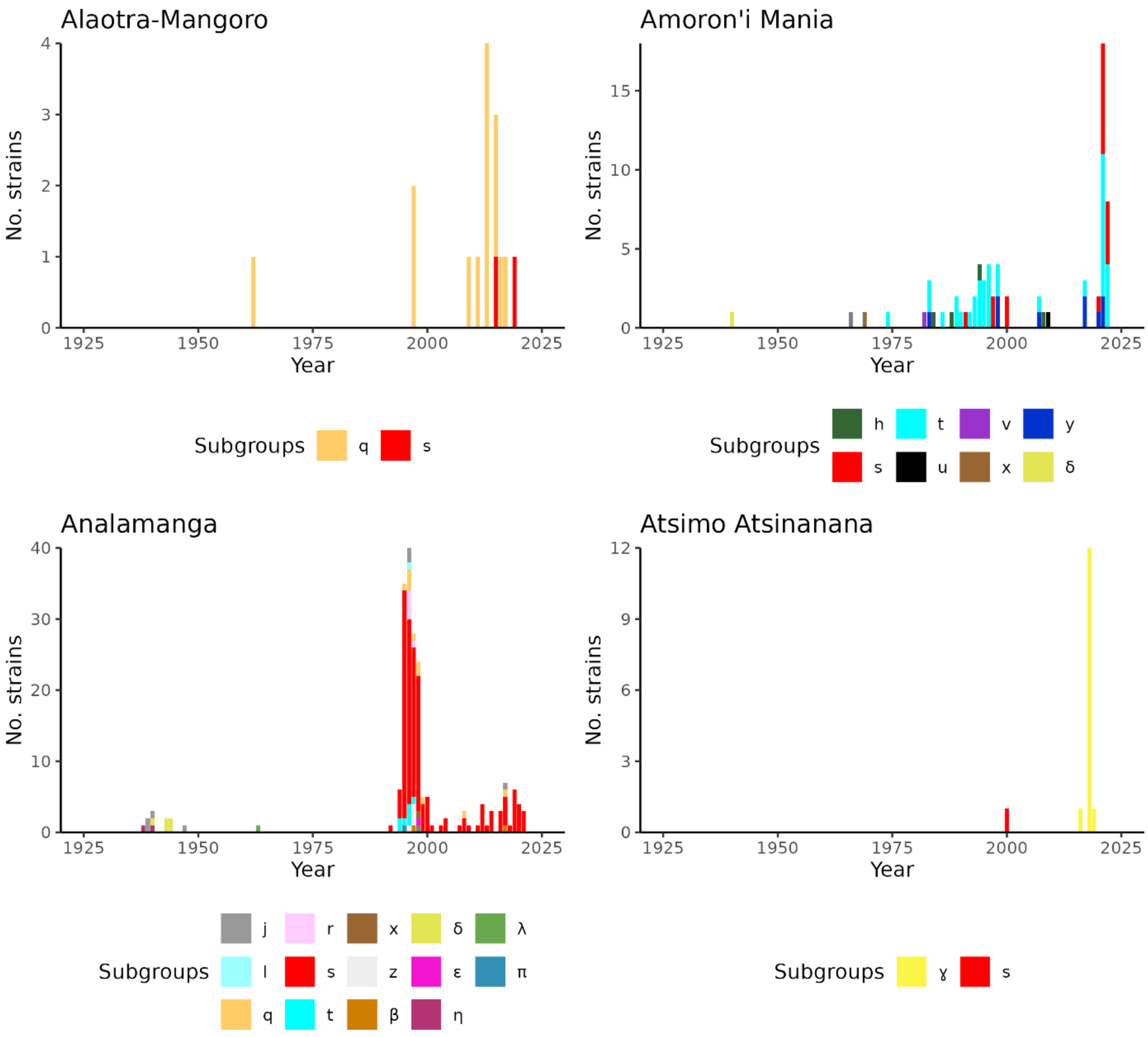

Supplementary Figure 3. Distribution of the *Y. pestis* strains isolated from Alaotra-Mangoro, Amoron'I Mania, Analamanga or Atsimo Atsinanana regions per year of isolation. Colors represent the genetic subgroup of the strains as indicated by the legend.

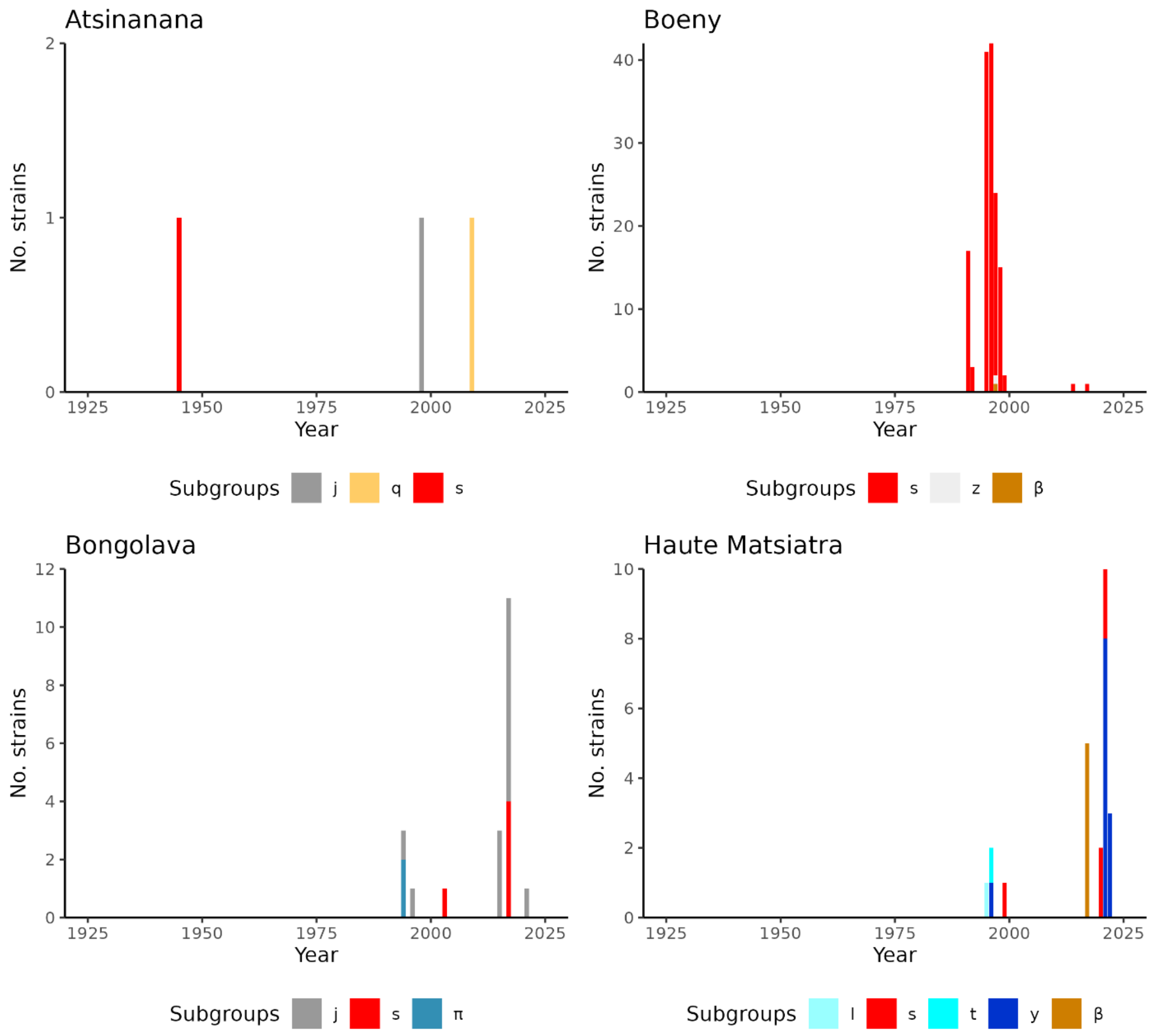

Supplementary Figure 4. Distribution of the *Y. pestis* strains isolated from Atsinanana, Boeny, Bongolava or Haute Matsiatra regions per year of isolation. Colors represent the genetic subgroup of the strains as indicated by the legend.

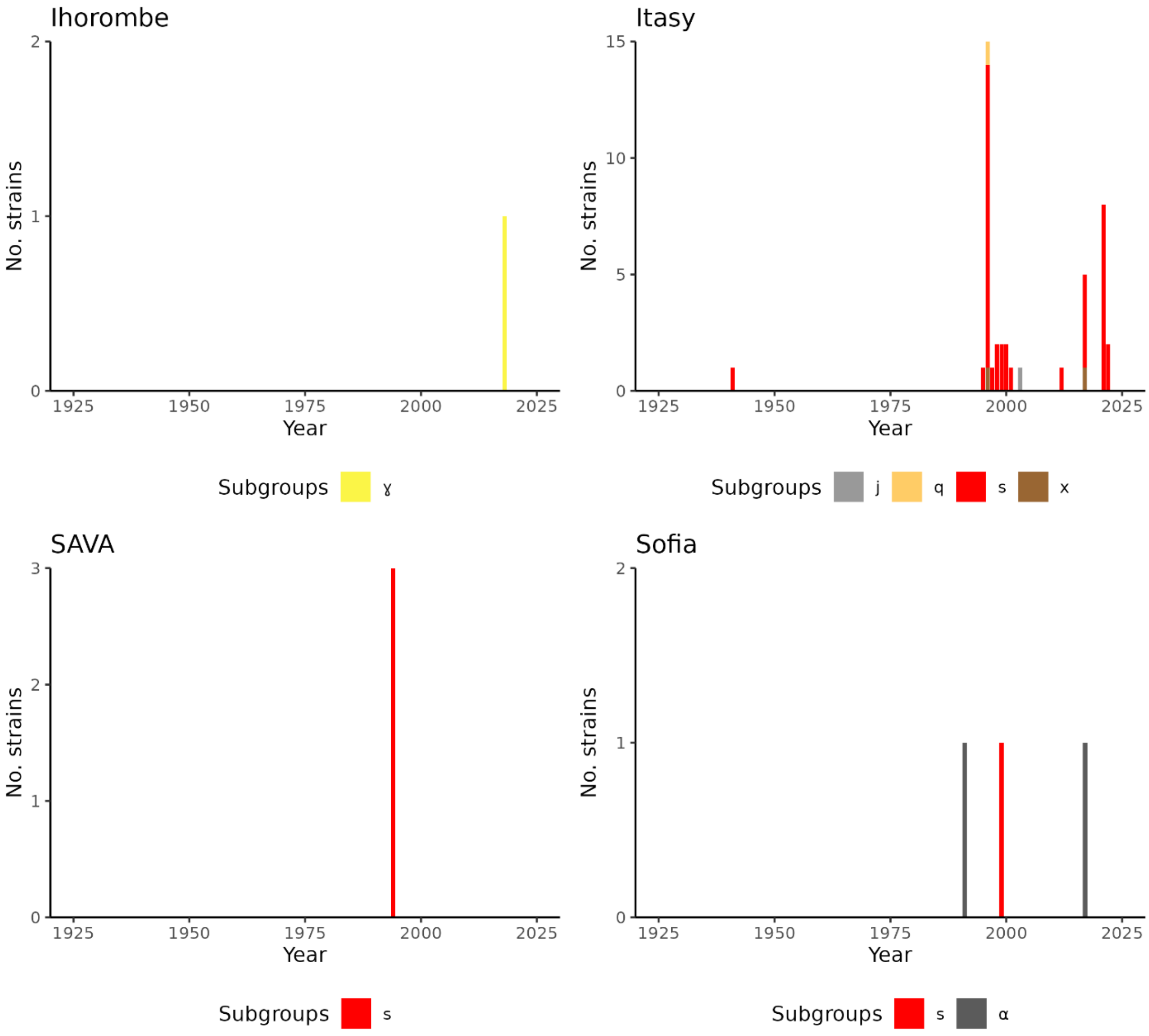

Supplementary Figure 5. Distribution of the *Y. pestis* strains isolated from Ihorombe, Itasy, SAVA or Sofia regions per year of isolation. Colors represent the genetic subgroup of the strains as indicated by the legend.

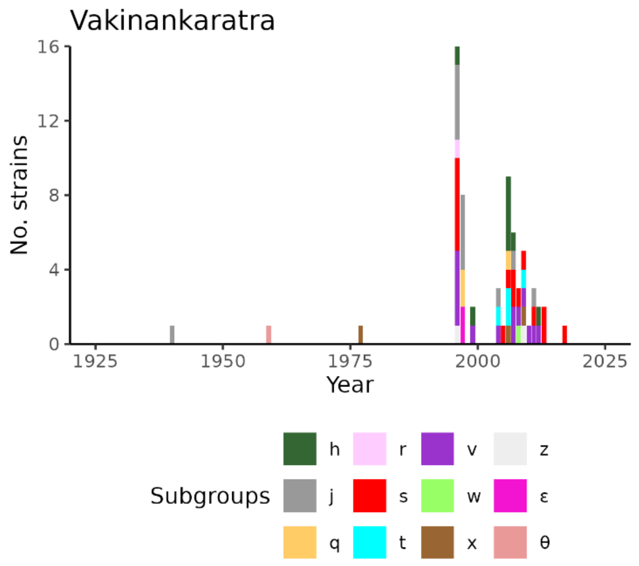

Supplementary Figure 6. Distribution of the *Y. pestis* strains isolated from Vakinankaratra region per year of isolation. Colors represent the genetic subgroup of the strains as indicated by the legend.
