# Supplementary material for: Evaluating the early diversification of Yersinia pestis and its phylogeographic expansion over 96 years of evolution in Madagascar
Source: Commun Biol. 2025 Nov 26;8:1705. doi: 10.1038/s42003-025-09109-1 (PMC12658076; doi:10.1038/s42003-025-09109-1)
Supplement: Supplementary file 2 — Description of Additional Supplementary Materials [file 42003_2025_9109_MOESM2_ESM.pdf]

Description of Additional Supplementary Files

File name: Supplementary Data 1

Description: SNP matrix behind the phylogeny

File name: Supplementary Data 2

Description: Metadata and source data behind the graphs in the paper

File name: Supplementary Data 3

Description: Source data behind Figure 3

File name: Supplementary Data 4

Description: List of the reads that needed trimming

File name: Supplementary Data 5

Description: Source data behind Figure 1b

File name: Supplementary Data 6

Description: Source data behind Figure 2b

File name: Supplementary Data 7

Description: tree file which is the source data for Supplementary Data 6
