# Supplementary material for: Evaluating the early diversification of Yersinia pestis and its phylogeographic expansion over 96 years of evolution in Madagascar
Source: Commun Biol. 2025 Nov 26;8:1705. doi: 10.1038/s42003-025-09109-1 (PMC12658076; doi:10.1038/s42003-025-09109-1)
Supplement: Supplementary file 5 — Reporting Summary [file 42003_2025_9109_MOESM5_ESM.pdf]

nature portfolio

Corresponding author(s): Lovasoa Nomena Randriantseheno

Last updated by author(s): Oct 3, 2025

Reporting Summary

Nature Portfolio wishes to improve the reproducibility of the work that we publish. This form provides structure for consistency and transparency in reporting. For further information on Nature Portfolio policies, see our Editorial Policies and the Editorial Policy Checklist.

Statistics

For all statistical analyses, confirm that the following items are present in the figure legend, table legend, main text, or Methods section.

n/a	Confirmed	
☒	☐	The exact sample size (*n*) for each experimental group/condition, given as a discrete number and unit of measurement
☒	☐	A statement on whether measurements were taken from distinct samples or whether the same sample was measured repeatedly
☒	☐	The statistical test(s) used AND whether they are one- or two-sided<br>*Only common tests should be described solely by name; describe more complex techniques in the Methods section.*
☒	☐	A description of all covariates tested
☒	☐	A description of any assumptions or corrections, such as tests of normality and adjustment for multiple comparisons
☒	☐	A full description of the statistical parameters including central tendency (e.g. means) or other basic estimates (e.g. regression coefficient) AND variation (e.g. standard deviation) or associated estimates of uncertainty (e.g. confidence intervals)
☒	☐	For null hypothesis testing, the test statistic (e.g. *F*, *t*, *r*) with confidence intervals, effect sizes, degrees of freedom and *P* value noted<br>*Give P values as exact values whenever suitable.*
☐	☒	For Bayesian analysis, information on the choice of priors and Markov chain Monte Carlo settings
☒	☐	For hierarchical and complex designs, identification of the appropriate level for tests and full reporting of outcomes
☒	☐	Estimates of effect sizes (e.g. Cohen's *d*, Pearson's *r*), indicating how they were calculated

Our web collection on statistics for biologists contains articles on many of the points above.

Software and code

Policy information about availability of computer code

Data collection	No software was used.
Data analysis	FastQC v0.11.9: used for quality control of the raw reads<br>MultiQC v1.15: used to compile and visualize the files outputted by FastQC<br>Trimmomatic v0.39: used to trim low quality reads and adaptors<br>BBduk in BBMap v39.01: used to filter out PhiX reads contaminating data<br>Minimap2 v2.28: used to map the reads against the reference genome<br>GATK v4.1.9: used to call variants<br>NUCmer v3.1: used by NASP to self-align the reference genome<br>NASP v1.2.1: used to filter out some SNPs<br>IQtree v2.1.2: used to infer the phylogenetic tree<br>iTol v7: used to visualize the phylogenetic tree<br>QGIS v3.22.11: used to map the genetic variants<br>Tempest v1.5.3: used to assess the presence of a temporal signal in our data<br>BEAST v1.8.4: used to run the Bayesian molecular dating analyses<br>Tracer: used to inspect the files outputted by BEAST<br>FigTree v1.4.4: used to visualize the dated phylogenetic tree

For manuscripts utilizing custom algorithms or software that are central to the research but not yet described in published literature, software must be made available to editors and reviewers. We strongly encourage code deposition in a community repository (e.g. GitHub). See the Nature Portfolio guidelines for submitting code & software for further information.

Data

Policy information about availability of data

All manuscripts must include a data availability statement. This statement should provide the following information, where applicable:

- Accession codes, unique identifiers, or web links for publicly available datasets
- A description of any restrictions on data availability
- For clinical datasets or third party data, please ensure that the statement adheres to our policy

Raw sequence data for all the strains newly sequenced in this study have been deposited in NCBI under Bioproject PRJNA1247195.

Research involving human participants, their data, or biological material

Policy information about studies with human participants or human data. See also policy information about sex, gender (identity/presentation), and sexual orientation and race, ethnicity and racism.

Reporting on sex and gender	NA
Reporting on race, ethnicity, or other socially relevant groupings	NA
Population characteristics	NA
Recruitment	NA
Ethics oversight	NA

Note that full information on the approval of the study protocol must also be provided in the manuscript.

Field-specific reporting

Please select the one below that is the best fit for your research. If you are not sure, read the appropriate sections before making your selection.

☒ Life sciences ☐ Behavioural & social sciences ☐ Ecological, evolutionary & environmental sciences

For a reference copy of the document with all sections, see nature.com/documents/nr-reporting-summary-flat.pdf

Life sciences study design

All studies must disclose on these points even when the disclosure is negative.

Sample size	No sample size calculation were performed but the sample size was chosen in order to best represent all plague foci in Madagascar during the study period.
Data exclusions	No data were excluded from the analyses.
Replication	No experimental analyses needing replication was undertaken in our study.
Randomization	Randomization is not relevant to our study, as no experimental group allocation was performed.
Blinding	Blinding is not relevant for our study because no experimental group allocation was performed.

Reporting for specific materials, systems and methods

We require information from authors about some types of materials, experimental systems and methods used in many studies. Here, indicate whether each material, system or method listed is relevant to your study. If you are not sure if a list item applies to your research, read the appropriate section before selecting a response.

Materials & experimental systems

n/a	Involved in the study
☒	☐ Antibodies
☒	☐ Eukaryotic cell lines
☒	☐ Palaeontology and archaeology
☒	☐ Animals and other organisms
☒	☐ Clinical data
☒	☐ Dual use research of concern
☒	☐ Plants

Methods

n/a	Involved in the study
☒	☐ ChIP-seq
☒	☐ Flow cytometry
☒	☐ MRI-based neuroimaging

Plants

Seed stocks

NA

Novel plant genotypes

NA

Authentication

NA
